# Positive and negative cooperativity of TNF and Interferon-γ in regulating synovial fibroblast function and B cell survival in fibroblast/B cell co-cultures

**DOI:** 10.1038/s41598-020-57772-7

**Published:** 2020-01-21

**Authors:** Torsten Lowin, Tareq M. Anssar, Marina Bäuml, Tim Classen, Matthias Schneider, Georg Pongratz

**Affiliations:** 1 Poliklinik, Funktionsbereich & Hiller Forschungszentrum für Rheumatologie, University Hospital Duesseldorf, D-40225 Duesseldorf, Germany; 2 Wilhelm Sander-NeuroOncology Unit and Department of Neurology, University Hospital of Regensburg, D-93053 Regensburg, Germany; 3 Laboratory of Experimental Rheumatology and Neuroendocrine Immunology, Dept. of Internal Medicine I, University of Regensburg, Regensburg, Germany; 4 Klinik für Orthopädie/Orthopädische Rheumatologie, St. Elisabeth-Hospital Meerbusch-Lank, D-40668 Meerbusch, Germany; 5 Department of Cardiology and Angiology I, University Heart Center Freiburg University, Freiburg, Germany

**Keywords:** Autoimmunity, Chemokines, Cytokines, Inflammation, Lymphocytes

## Abstract

Synovial fibroblasts (SF) were reported to produce B cell activating factor (BAFF) in response to stimulation with interferon-γ (IFN-γ) or tumor necrosis factor (TNF). However, the influence of these pro-inflammatory cytokines on other receptors/ligands of the TNF superfamily or associated cytokine receptors in SF has not been investigated yet. Here we show the differential regulation of BAFF (CD257), Fn14 (CD266), TACI (CD267), BAFF-R (CD268), BCMA (CD269), CD40 ligand (CD40L, CD154), IFN-γR (CD119), Leptin receptor (ObR, CD295), VCAM-1 (CD106) and membrane TGF-β in isolated SF and the impact of IFN-γ/TNF co-incubation on proliferation, IL-6 and IL-8 production. In addition, the impact of differentially stimulated SF on B cell survival in co-cultures was assessed. Surface cytokines and cytokine receptors were detected by flow cytometry. Soluble cytokine receptors and cytokines were quantified by ELISA. Proliferation was assessed by cell titer blue. Murine B cell survival in fibroblast/ B cell co-cultures was determined by annexin V/propidium iodide staining and flow cytometry. IFN-γ together with TNF synergistically and significantly increased the cell surface levels of BAFF, Fn14, TACI, BAFF-R, BCMA, CD40L, ObR and IFN-γR in rheumatoid arthritis SF after 72 h incubation. Soluble BAFF was only induced by IFN-γ and inhibited by TNF. Addition of TWEAK had no influence on proliferation or IL-8 production but decreased TNF-induced IL-6 production, whereas APRIL, BAFF and leptin did not modulate TNF or TNF/IFN-γ-induced proliferation or cytokine production. Proliferation was increased by TNF and further enhanced by the addition of IFN-γ. In co-culture experiments, SF stimulated with TNF/IFN but not TNF or IFN-γ alone increased shedding of VCAM-1 and expression of membrane TGFβ, which was associated with reduced survival of murine B cells. IFN-γ and TNF regulate the expression of TNF family member cytokines and associated receptors. Ligation of IFN-γR and Fn14 under pro-inflammatory conditions modulated IL-6/IL-8 production and proliferation. In B cell/SF co-cultures, the combination of TNF/IFN reduced B cell survival possibly via enhanced VCAM-1 shedding and/or increased TGF-β production. IFN-γ is necessary for the observed effects on B cell survival and SF cytokine production and emphasizes its anti-inflammatory role in rheumatoid arthritis.

## Introduction

In rheumatoid arthritis (RA), interferon-γ (IFN-γ) levels are increased in synovial fluid and concomitantly, synovial fluid mononuclear cells show increased IFN-γ mRNA levels^1,2^. IFN-γ has a dual role in chronic inflammation since it does increase the presentation of antigens via induction of MHC class II molecules in synovial fibroblasts (SF) and foster chemokine production^3,4^. Conversely, IFN-γ counteracts matrix metalloprotease production by IL-1β and increases production of the IL-18 scavenger IL-18 binding protein in SF^5,6^. In addition, several studies already evaluated IFN-γ as an anti-arthritic therapy and reported a modest beneficial effect on pain and joint swelling^7–10^. These findings are further emphasized by the fact that IFN-γ receptor (IFN-γR, CD119) knockout mice are highly susceptible to the development of arthritis^11^. In rheumatoid arthritis synovial fibroblasts (RASF), IFN-γ has been found to increase the expression of B cell activating factor (BAFF, CD257)^12^. BAFF is a member of the TNF superfamily of ligands and receptors and is an important cytokine for the survival of autoreactive B cells^13^. Besides BAFF, SF express the related molecules B cell maturation antigen (BCMA, CD269), BAFF receptor (CD268), Fn14 (TWEAK receptor, CD266) transmembrane activator and CAML interactor (TACI, CD267) and possibly membrane-bound APRIL (a proliferation inducing ligand, CD256)^14–16^. Although some effects of APRIL and TWEAK (TNF-related weak inducer of apoptosis) on cytokine production and proliferation in SF have been described, it is still unclear how these ligands influence SF function^14,15,17^. In addition, the effects of pro-inflammatory cytokines on the expression of CD266, CD267, CD268, CD269 and CD154 have not been investigated. In this study, we shed light on the regulation of these surface receptors by cytokines, which play an important role in RA (TNF and IFN-γ) and further provide evidence that SF pre-stimulated with these cytokines differentially influence B cell survival. In addition we investigated the surface levels of ObR (leptin receptor) and influence of leptin on the effects of TNF and IFN-γ, since leptin has been shown to enhance IFN-γ signaling by activating JAK/STAT pathways^18^.

## ResultsTNF and IFN-γ Regulate Cell Surface Cytokines and Cytokine Receptors

**Figure 1:**
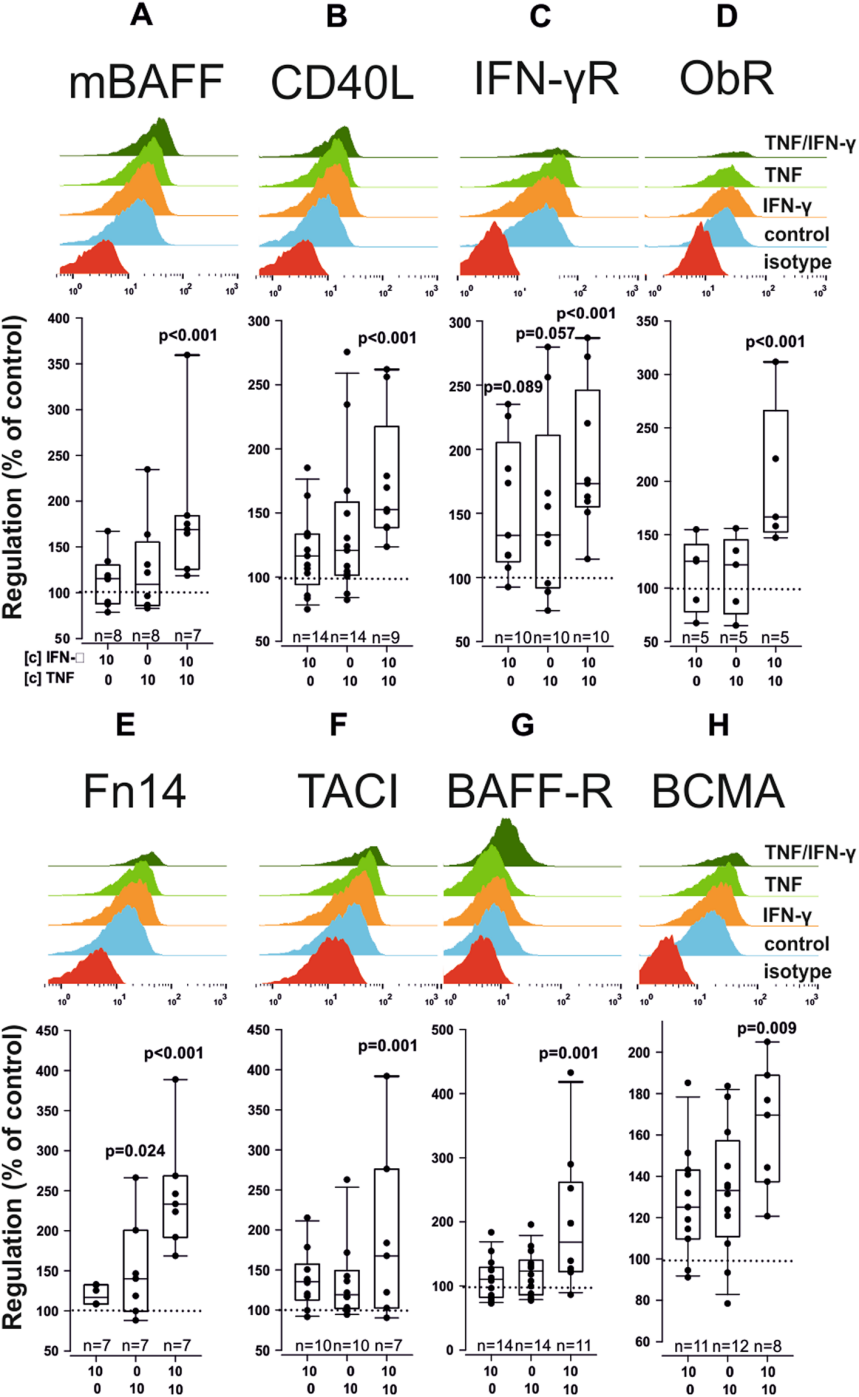
Cell surface levels of membrane-bound BAFF (**A**), CD40L (**B**), IFN-γR (**C**), ObR (**D**), Fn14 (**E**), TACI (**F**), BAFF-R (**G**) and BCMA (**H**) after stimulation with given cytokines for 72 h. The upper panels depict exemplary histograms after flow cytometric analyses. All FACS measurements were normalized to the geometric mean of the unstimulated control value, which was set at 100% (dotted line). P-values are given in the graph. The general linear model with Bonferroni correction was used for all comparisons versus control.

We determined the cell surface levels of BAFF, TACI, BCMA, BAFF-R, CD40L, Fn14, INF-γR and ObR under the influence of TNF (10 ng/ml), and IFN-γ (10 ng/ml). Whereas INF-γ alone had no significant effect on the proteins investigated, we found the combination of IFN-γ and TNF to be a strong stimulus that robustly increased membrane (m)BAFF (185% ± 80%; p < 0.001; Fig. 1A), CD40L (174% ± 51%; p < 0.001; Fig. 1B), IFN-γR (190% ± 54%; p < 0.001; Fig. 1C), ObR (200% ± 68%; p < 0.001; Fig. 1D), Fn14 (246% ± 71%; p < 0.001; Fig. 1E), TACI (190% ± 108%; p = 0.001; Fig. 1F), BAFF-R (264% ± 246%; p = 0.001; Fig. 1G) and BCMA (202% ± 115%; p = 0.009; Fig. 1H) in RASF. However, TNF alone increased the surface levels of Fn14 (151% ± 63%; p = 0.032; Fig. 1E) and per statistical trend IFN-γR (147% ± 70%; p = 0.057; Fig. 1C).

**Figure 2:**
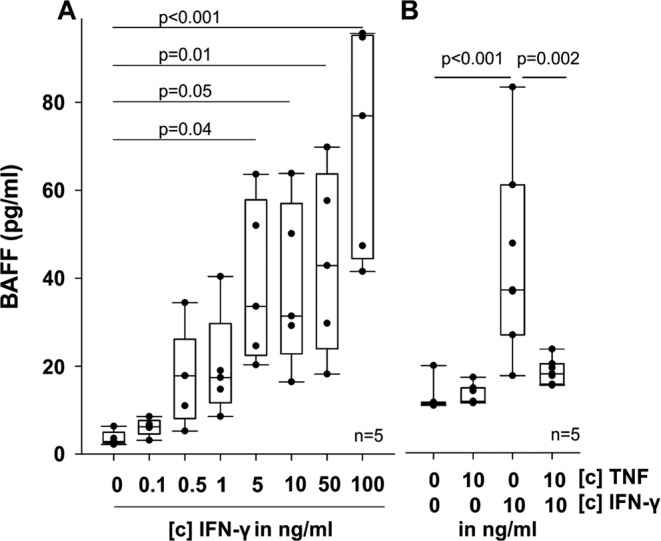
BAFF production by RASF in 48 h. (**A**) IFN-γ induced BAFF production by RASF. (**B**) Influence of TNF alone and in combination with IFN-γ on BAFF release. P-values are given in the graph. The general linear model with Bonferroni correction was used for all comparisons versus control.

IFN-γ induces soluble BAFF, which in turn is inhibited by TNF. With the exception of IFN-γR, all TNF family member proteins investigated in this study are also shedded from the cell surface^19,20^, and therefore we assessed the amount of soluble BAFF, APRIL, CD40L, BCMA and TACI. In RASF stimulated with TNF, IFN-γ, or a combination of both cytokines for 72 h, no soluble APRIL, CD40L, BCMA and TACI was detected (data not shown). In contrast, BAFF production was dose-dependently induced by IFN-γ (Fig. 2A) and inhibited by addition of TNF (p = 0.002; Fig. 2B).

### IFN-γ and TWEAK influence RASF proliferation

**Figure 3:**
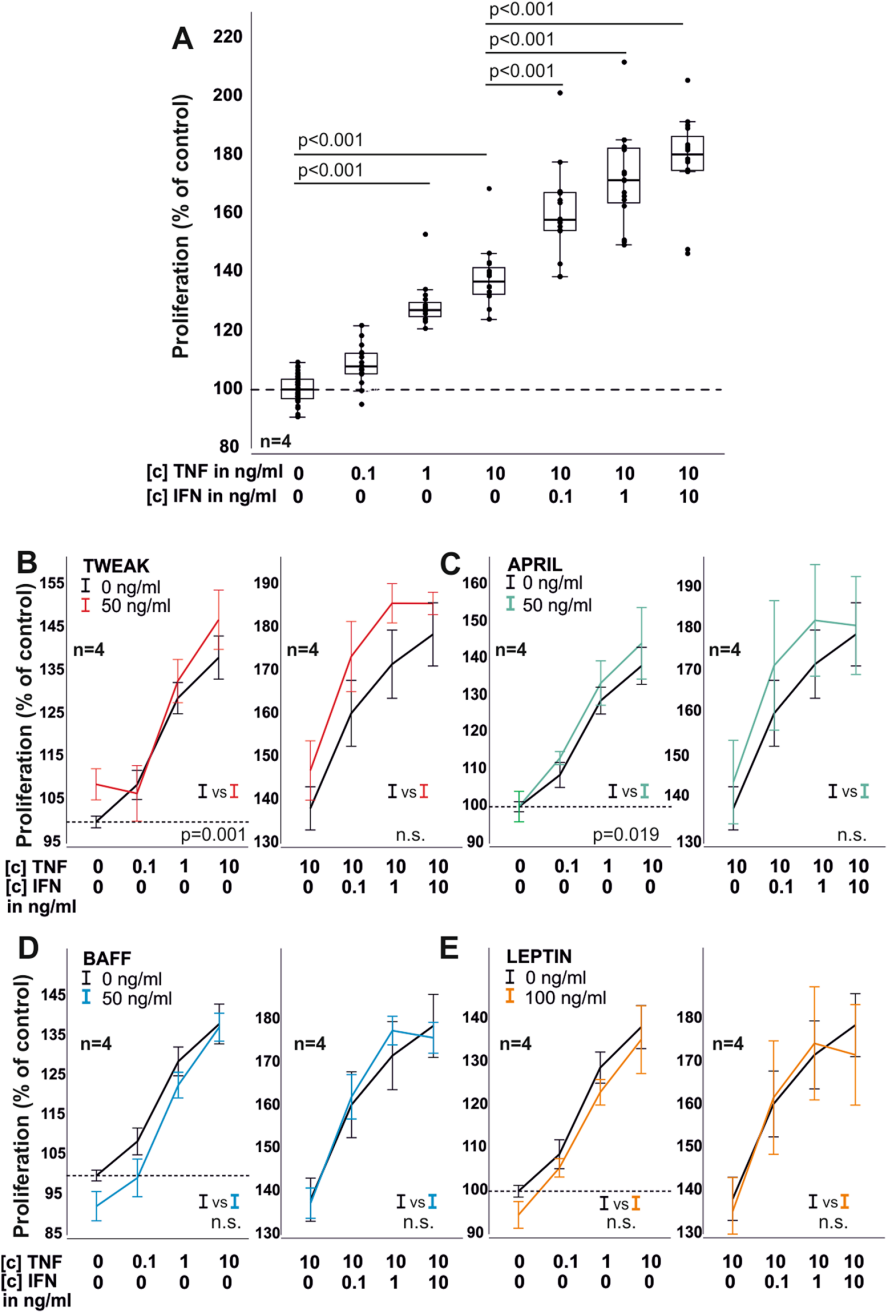
Proliferation of RASF in 72 h under the influence of TNF, IFN-γ, TWEAK, APRIL, BAFF and leptin. (**A**) Proliferation in response to TNF and TNF combined with IFN-γ. (**B**) same as A) but with the addition of TWEAK (red curves). (**C**) same as A) but with the addition of APRIL (green curves). (**D**) same as A) but with the addition of BAFF (blue curves). (**E**) same as A) but with the addition of leptin (orange curves). P-values are given in the graph. Each dot represents one well of a 96 well plate. The general linear model with Bonferroni correction was used for comparisons in A) and ANOVA for all other comparisons (**B**-**E**). The dotted line represents the control value which was set to 100%. Data in (**B**-**E**) are presented as line blots and depict mean values. Whole curves were compared using one-way ANOVA analyses.

Since we found TNF/IFN-γ to upregulate Fn14, BCMA, TACI, BAFF-R, IFN-γR and ObR, we next investigated how ligation of these receptors with TWEAK (Fn14), BAFF (BAFF-R = TACI»BCMA), APRIL (BCMA = TACI), IFN-γ (IFN-γR) and leptin (ObR) influences RASF proliferation and the cytokine-induced production of IL-6 and IL-8. Proliferation of RASF was significantly increased by the addition of TNF ([c] 1 ng/ml, 128% ± 7.1; p < 0.001; [c] 10 ng/ml, 138% ± 11; p < 0.001) and further enhanced by the addition of IFN-γ as compared to TNF 10 ng/ml alone ([c] 0.1 ng/ml, 161% ± 17; p < 0.001; [c] 1 ng/ml, 172% ± 17; p < 0.001; [c] 10 ng/ml, 178% ± 16; p < 0.001; Fig. 3A). TWEAK and APRIL exerted a positive effect on TNF-induced proliferation (p = 0.001 and p = 0.019, respectively; Fig. 3B,C) while BAFF and leptin had no influence on proliferation (Fig. 3D,E).

### IFN-γ and TWEAK influence RASF cytokine production

**Figure 4:**
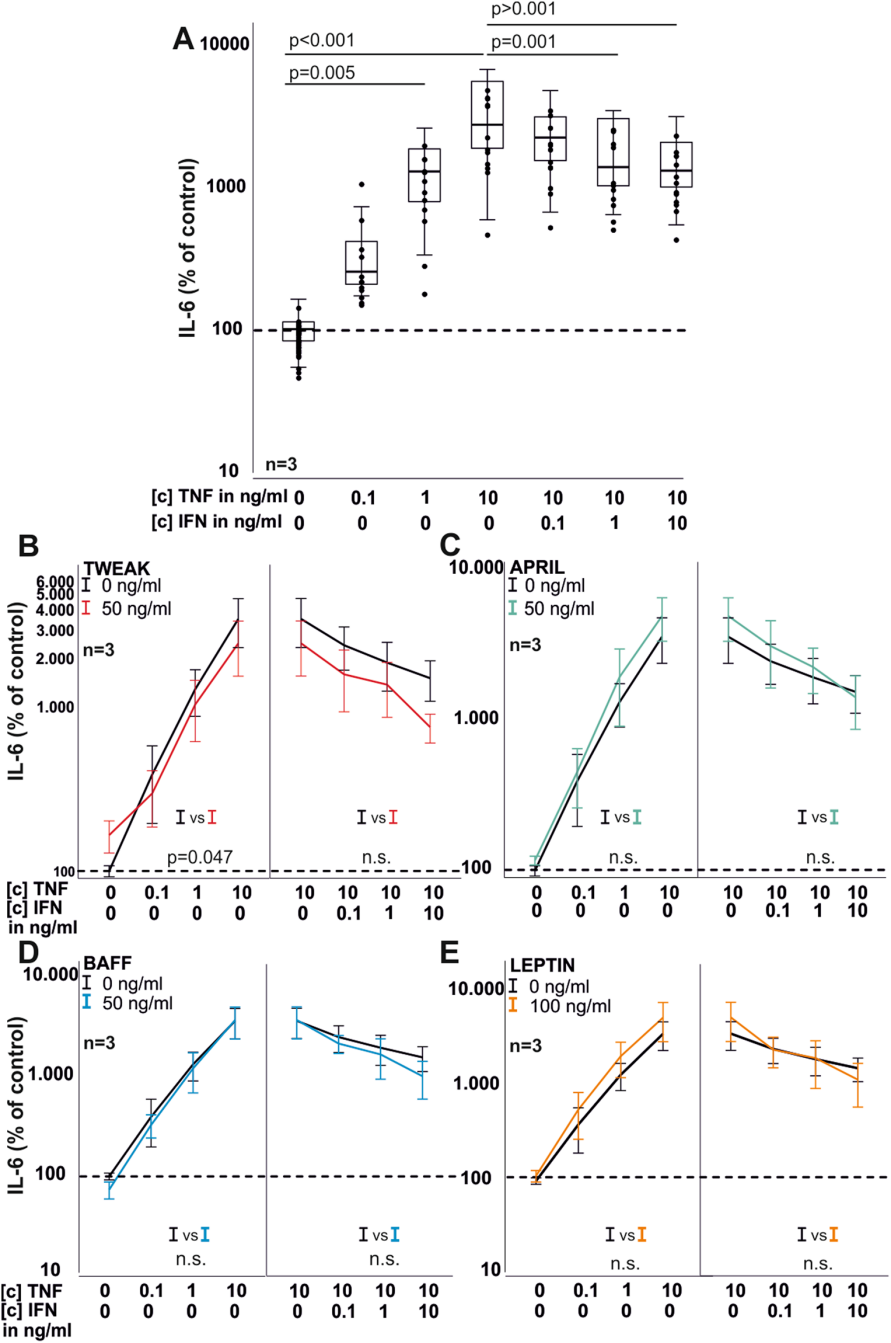
IL-6 production by RASF in 72 h under the influence of TNF, IFN-γ, TWEAK, APRIL, BAFF and leptin. (**A**) IL-6 production in response to TNF and TNF combined with IFN-γ. (**B**) same as A) but with the addition of TWEAK (red curves). (**C**) same as A) but with the addition of APRIL (green curves). (**D**) same as A) but with the addition of BAFF (blue curves). (**E**) same as A) but with the addition of leptin (orange curves). Each dot represents one well of a 96 well plate. The general linear model with Bonferroni correction was used for comparisons in A) and ANOVA for all other comparisons (**B**-**E**). The dotted line represents the control value which was set to 100%. Data in (**B**-**E**) are presented as line blots and depict mean values. Whole curves were compared using one-way ANOVA analyses.

**Figure 5:**
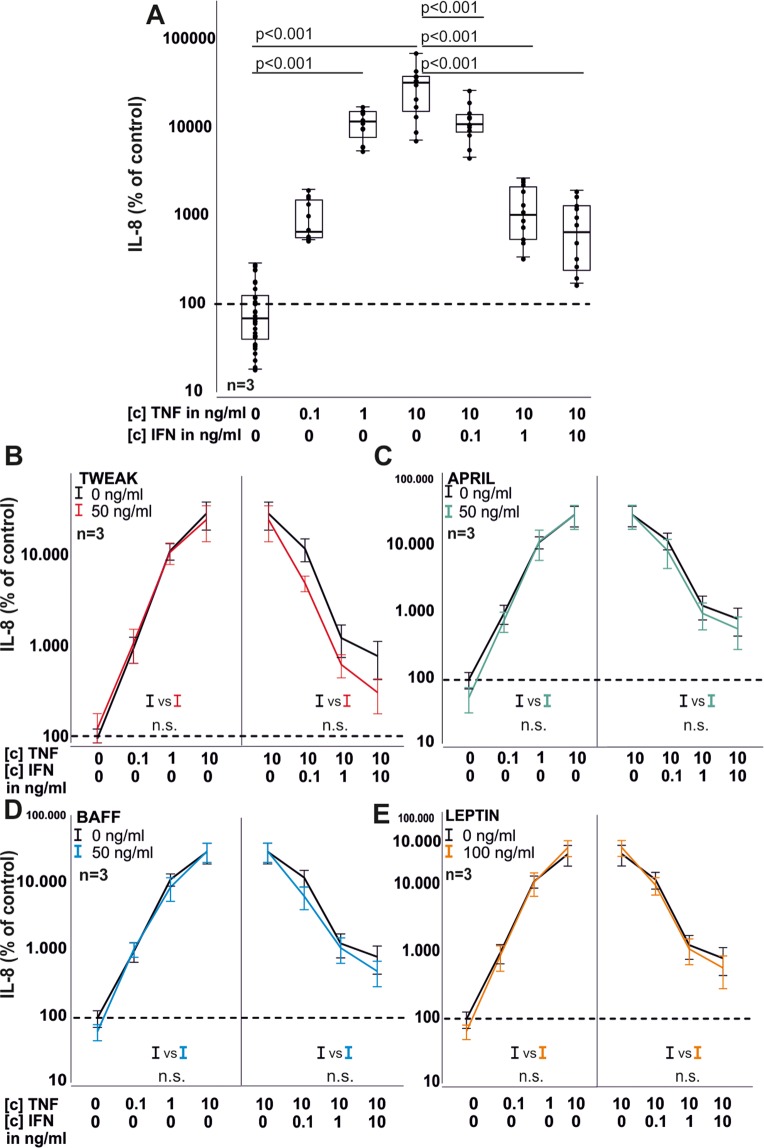
IL-8 production by RASF in 72 h under the influence of TNF, IFN-γ, TWEAK, APRIL, BAFF and leptin. (**A**) IL-8 production in response to TNF and TNF combined with IFN-γ. (**B**) same as A) but with the addition of TWEAK (red curves). (**C**) same as A) but with the addition of APRIL (green curves). (**D**) same as A) but with the addition of BAFF (blue curves). (**E**) same as A) but with the addition of leptin (orange curves). Each dot represents one well of a 96 well plate. The general linear model with Bonferroni correction was used for comparisons in A) and ANOVA for all other comparisons (**B**–**E**). The dotted line represents the control value which was set to 100%. Data in (**B**–**E**) are presented as line blots and depict mean values. Whole curves were compared using one-way ANOVA analyses.

Next, IL-6 and IL-8 production of RASF were investigated under the influence of TNF and IFN-γ together with TWEAK, APRIL, BAFF and leptin. As expected, TNF dose-dependently increased IL-6 ([c] 1 ng/ml, 1334% ± 739%; p = 0.005, [c] 10 ng/ml, 3616% ± 2096%, p < 0.001; Fig. 4A) and IL-8 ([c] 1 ng/ml, 11617% ± 4146%; p < 0.001; [c] 10 ng/ml 29906% ± 17668%, p < 0.001 for IL-8, Fig. 5A) production. Addition of IFN γ antagonized TNF (10 ng/ml) –induced IL-6 ([c] 1 ng/ml, 19447% ± 1123%; p = 0.001; [c] 10 ng/ml, 1558% ± 755%, p < 0.001 Fig. 4A) and IL-8 ([c] 0.1 ng/ml, 12342% ± 5999%; p < 0.001; [c] 1 ng/ml, 1289% ± 870%, p < 0.001; [c] 10 ng/ml, 816% ± 633%, p < 0.001, Fig. 5A) production, respectively. TWEAK (p = 0.047, Fig. 4B) but not BAFF (Fig. 4D), APRIL (Fig. 4C) or leptin (Fig. 4E) reduced TNF-induced IL-6 production. The TNF-induced or TNF/IFN-γ-induced IL-8 production was not modulated by TWEAK, APRIL, BAFF or leptin (Fig. 5B–E).

### TNF and IFN-γ stimulated RASF modulate B cell survival

**Figure 6:**
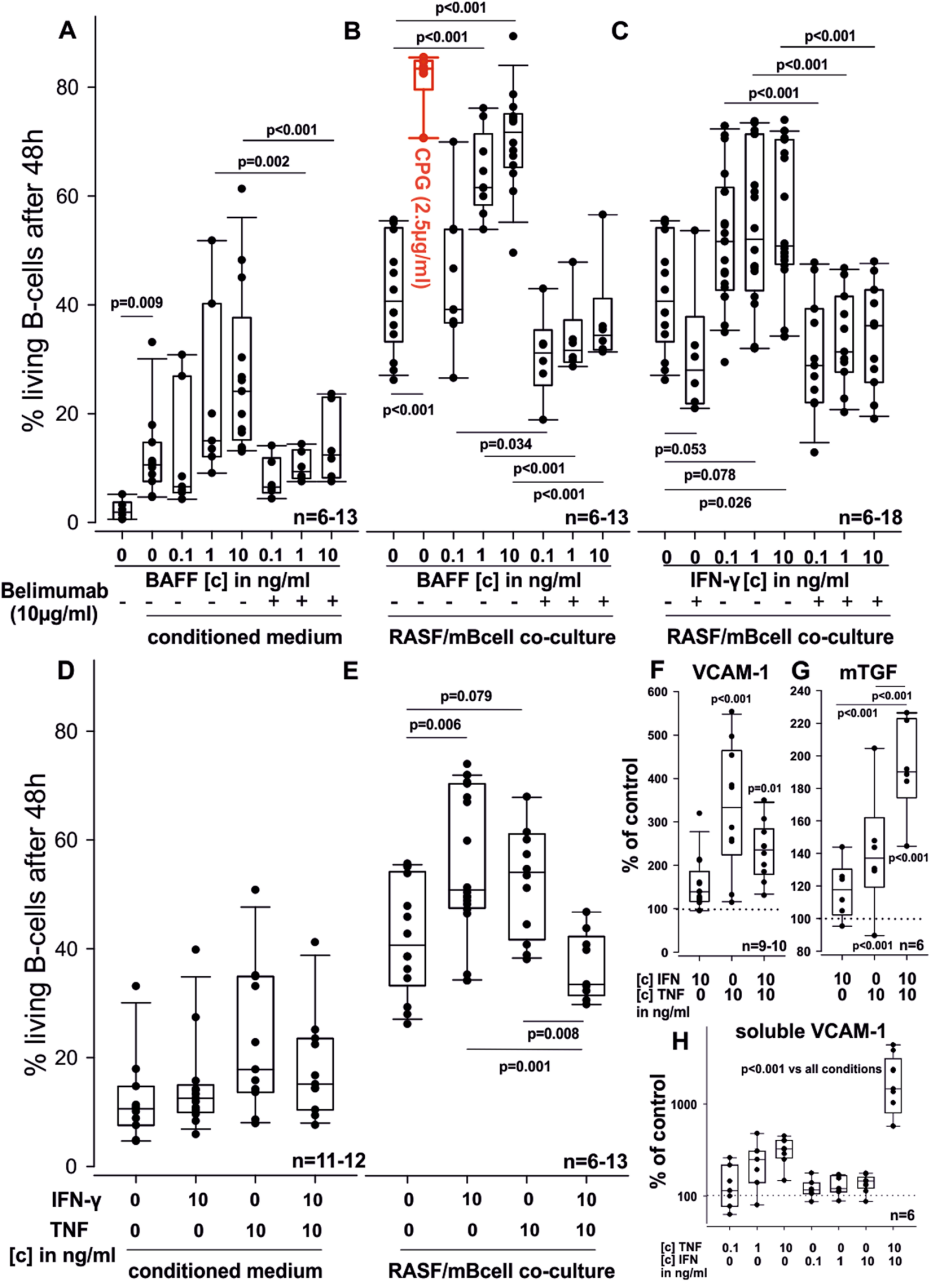
Murine B cell survival alone and in RASF co-culture, VCAM-1 and TGF-β levels on RASF. (**A**,**D**) B cell survival in conditioned medium. B cells were transferred in conditioned medium generated from RASF cultured for 48 h with or without BAFF, belimumab, TNF or IFN-γ. (**B**,**C**,**E**) B cell/RASF co-culture. All given cytokines or belimumab were added to RASF 48 h prior to B cell addition. CPG (red) was used as a positive control. (**F**,**G**) Flow cytometric cell surface staining of VCAM-1(F) and TGF-β (G) on RASF. (**H**) Soluble VCAM-1 in RASF supernatants measured by ELISA. RASF were stimulated with given cytokines for 72 h (**G**,**H**). P-values are given in the graph. The general linear model with Bonferroni correction was used for all comparisons. The dotted line represents the control value which was set to 100% (**F**–**H**).

Since cytokines of the TNF family are broadly involved in B cell homeostasis, we aimed to clarify whether RASF stimulated with TNF or IFN-γ differentially affect B cell survival. Since we used murine B cells (mBcells) on human fibroblasts for these experiments, we first showed that human BAFF has an impact on murine B cells and found that conditioned medium from RASF stimulated for 48 h with human BAFF (0.1 ng/ml–10 ng/ml) increased murine B cell survival in RASF/B cell co cultures (Fig. 6A). Conditioned medium also increased survival in B cell monocultures (p = 0.009, B cells alone vs B cells in conditioned medium, Fig. 6A). The pro-survival effect was mediated to a great extent by BAFF, since addition of the BAFF neutralizing antibody belimumab at 10 µg/ml decreased survival back to control values (p < 0.001, [c] IFN-γ 10 ng/ml, Fig. 6A). Similar results were obtained in co-culture experiments in which murine B cells were co-cultivated with BAFF-stimulated RASF for 48 h (Fig. 6B). BAFF (1 ng/ml and 10 ng/ml) increased murine B cell survival (p < 0.001) also in coculture with RASF and this effect was inhibited by addition of belimumab (p < 0.001). Since IFN-γ induces the production of BAFF in RASF, we speculated that IFN-γ pre-treated RASF increase mBcell survival by enhanced BAFF production. Indeed, belimumab inhibited the pro-survival effects of IFN-γ treated RASF on B cell survival (p < 0.001, Fig. 6C). We then tested how RASF stimulated with TNF alone or in combination with IFN- γ affects murine B cell survival. Conditioned medium from RASF treated with TNF, IFN-γ or a combination did not enhance mBcell survival (Fig. 6D). Conversely, in RASF/mBcell co-culture, TNF (p = 0.079) or IFN-γ (p = 0.006) alone increased mBcell survival, while a combination of both decreased B cell survival (p = 0.008 vs TNF alone and p = 0.001 vs IFN-γ alone, Fig. 6E). These data suggest that expression of a specific, membrane-bound survival molecule is altered in response to TNF/IFN-γ in RASF. Since we found membrane-bound BAFF and CD40L, both factors that would augment B cell survival^13,21^, to increase with TNF/IFN-γ, these molecules are unlikely candidates for the decreased survival of B cells cultivated together with TNF/IFN-γ-stimulated RASF. We suspected a different protein on RASF to be responsible for decreased B cell survival. One important survival factor for B cells is VCAM-1^22^ and therefore, we investigated how this adhesion molecule is regulated under our culture conditions. We found that TNF (+332% ± 216%, p < 0.001) and TNF/IFN-γ (+214% ± 77%, p = 0.01) induce significant cell surface expression of VCAM-1 (Fig. 6F). Since VCAM-1 is shedded from the cell surface by the enzyme ADAM17^23^, we also determined soluble VCAM-1 levels. We found that only TNF/IFN-γ strongly induced soluble VCAM-1 (1464 pg/ml ± 675 pg/ml vs 90 pg/ml ± 77 pg/ml (control); + 2286% ± 1421%, p < 0.001, Fig. 6H). While differential VCAM-1 levels on RASF might alter B cell survival, RASF also produce transforming growth factor beta (TGF-β), a cytokine reported to influence B cell maturation, homeostasis, activation and most importantly, to promote apoptosis^24–27^. Consequently, we also assessed cell surface levels of this cytokine and found that TNF (+141% ± 37%, p < 0.001) and, even more pronounced, TNF/IFN-γ (+193% ± 30%, p < 0.001) up-regulated membrane-bound TGF-β (Fig. 6G). TNF/IFN-γ induced significantly higher levels of membrane-bound TGF-β as compared to TNF or IFN-γ alone (p < 0.001, Fig. 6G).

## Discussion

In this study, we demonstrated that the combination of TNF and IFN-γ up-regulated the surface expression of the cytokine receptors TACI, BCMA, BAFF-R, Fn14, IFN-γR, ObR, CD40L and also the membrane-bound form of the B cell survival cytokine BAFF in RASF. IFN-γ further enhanced TNF-induced proliferation of RASF while inhibiting IL-6 and IL-8 production. On the other hand, TNF reduced IFN-γ-induced BAFF production. Activation of Fn14 with TWEAK increased TNF-induced proliferation while it decreased TNF-induced IL-6 production by RASF. In RASF/murine B cell co-cultures we found RASF to strongly support B cell survival and this was further enhanced by the addition of BAFF. In addition, RASF stimulated with IFN-γ or TNF increased B cell survival while a combination of both reduced survival. Survival might be linked to the expression and increased shedding of VCAM-1 and membrane-bound TGF-β which are up-regulated by TNF/IFN-γ. To our knowledge, this is the first study that investigated the regulation of surface levels of BAFF, BAFF-R, BCMA, TACI, Fn14, CD40L, ObR and IFN-γR on isolated RASF. In contrast to results from Nakajima *et al*., we detected BAFF-R on the cell surface of RASF which was increased by TNF/IFN-γ^28^. BCMA and TACI have already been shown to be expressed on RASF and rat arthritic fibroblasts, respectively, but their regulation has not been investigated^14,15^. In line with data from Kamijo *et al*. we detected Fn14 on RASF and found that TNF alone and TNF/IFN-γ are inducers of its expression^29^. Since we used IFN-γ as one major stimulus, we were also interested in the regulation of its receptor and showed an increase in response to TNF/IFN-γ. This is in line with results from Alvaro-Gracia *et al*. who demonstrated an increase of IFN-γ binding after TNF stimulation on RASF^30^. Binding of IFN-γ to its receptor is usually associated with a downregulation of IFN-γR, but this does not occur in fibroblasts^31,32^. We also assessed the influence of IFN-γ and TNF on leptin recepter (ObR) expression and found this combination to increase ObR surface levels. Like IFN-γR, ObR signaling is initiated by JAK/STAT activation and some effects of IFN-γ are potentiated by leptin which has been associated with disease activity in RA^18,33^. In addition, TNF has been shown to increase surface levels of ObR by the activation of protein kinase C^34^. Functionally, leptin has already been shown to increase IL-6 levels in SF but we did not observe a potentiation of TNF or TNF/IFN-γ effects in RASF by leptin. In a next step, we investigated the expression of molecules important for B cell survival and activation in RASF. An indispensable co-stimulatory protein to activate B cells in a T cell dependent manner is CD40L, whose expression by RASF in contrast to CD40, has not been demonstrated directly. The ability of RASF to activate B cells and induce antibody class switching, however, suggests CD40L expression^35–37^. Indeed, we found CD40L on the surface of RASF and its levels were augmented by TNF/IFN-γ which might support B cell activation by RASF under these conditions. Another important molecule for B cell function is BAFF, which enhances the survival and class switching of (autoreactive) B cells^13,37^. We found an increase of membrane-bound BAFF on RASF after stimulation with TNF/IFN-γ, whereas soluble BAFF was induced only by IFN-γ and inhibited by TNF. This somewhat contrasts results from Reyes *et al*. that showed increased BAFF levels after TNF stimulation but supports data from Alsaleh *et al*. who demonstrated reduced BAFF expression in response to TNF^38,39^. However, reported TNF-induced BAFF levels in the study by Reyes were extremely low (below 10 pg/ml) and we considered such low amounts as baseline level in our study since control values for BAFF in unstimulated RASF were between 0 and 10 pg/ml. Since we found an upregulation of cytokine receptors TACI, BAFF-R, BCMA, ObR and IFN-γR after stimulation with TNF/IFN-γ, we next investigated whether ligands for these receptors have any impact on proliferation or cytokine production by RASF. We found IFN-γ to further enhance TNF-induced proliferation with a concomitant reduction in IL-6 and IL-8 production. With respect to RASF proliferation this contrasts results from Alvaro-Garcia *et al*., who showed reduced proliferation when TNF was combined with IFN-γ^40^. This might be due to different culture conditions since we used RPMI medium with only 2% FCS while the above mentioned study used DMEM with 10% FCS. We found only minimal basal proliferation using our culture conditions, with strong induction of proliferation upon addition of cytokines. Using less stringent culture conditions, where basal proliferation is already significant, IFN-γ might exert different effects. Additionally, Alvaro-Garcia *et al*. demonstrated that IFN-γ reduced collagenase expression in RASF, which supports our observations regarding IL-6 production, since collagenase and IL-6 are both induced by TNF and employ overlapping transcriptional regulation of mRNA synthesis^41,42^. In line with the inhibitory effects of IFN-γ on RASF, studies from Schlaak *et al*. and Ohta *et al*. showed reduced IL-6 production when IL-4 was combined with IFN-γ and diminished IL-8 production when IFN-γ and TNF were added together, respectively^43,44^. Our results demonstrated a dual role of IFN-γ on inflammation: It cooperates with TNF to up-regulate membrane-bound cytokines and receptors on RASF while concomitantly decreasing the release of cytokines IL-6 and IL-8. IFN-γ is usually produced in a TH1-biased environment during the development of the adaptive immune response and in this situation it might be detrimental to attract innate immune cells such as neutrophils that cannot support a T cell/ B cell response. In fact, it has been demonstrated that IFN-γ priming of macrophages before an LPS challenge reduced the production of mediators necessary for leukocyte recruitment^45^.

We further examined the impact of TWEAK, APRIL, BAFF and leptin on TNF and TNF/IFN-γ induced proliferation and IL-6/IL-8 production. TWEAK supported TNF but not TNF/IFN-γ induced proliferation and reduced IL-6 production only when induced by TNF but not by TNF/IFN-γ. While Yamana *et al*. also found TWEAK to reduce TNF-induced proliferation and IL-6 synthesis in RASF, TWEAK increased TNF-induced proliferation in our study^17^. In contrast to Yamana *et al*., we incubated RASF for 72 h instead of 48 h and included different TNF concentrations. In addition, like mentioned above with IFN-γ, we used only 2% FCS in culture medium for proliferation assays, which might change the effects of TWEAK from inhibitory to stimulatory. TWEAKs ability to promote proliferation is in line with *in vivo* results, where TWEAK has been shown to stimulate wound-healing^46,47^. APRIL modestly increased TNF-induced proliferation but not IL-6 or IL-8 production. In contrast, data from Nagatani *et al*. showed that APRIL fosters IL-6 mRNA expression in RASF, but this study investigated the effects of APRIL without concomitant stimulation with TNF^15^. In addition, the same study demonstrated significant APRIL mRNA production by RASF, which was not confirmed on protein level of soluble APRIL by our results or by Alsaleh *et al*.^37^. However, APRIL might be present in a membrane-bound form, which has been demonstrated on the surface of macrophages^48^. Overall, the effects of APRIL on RASF are weak and might only be relevant before the adaptive immune response takes over. BAFF exerted no influence on proliferation, IL-6 and IL-8 production of RASF. In HT1080 fibrosarcoma cells however, BAFF serves as an autocrine stimulator of basal NFκB activity by binding to intracellular BAFF-R^49^. Since we only employed surface staining of BAFF-R, there might still be a receptor pool located intracellularly. Addition of exogenous BAFF likely activates membrane-bound BAFF-R exclusively, which does not exclude a specialized role of intracellular BAFF in RASF, which needs to be defined in future studies.

Since we found synergistic and antagonistic effects of IFN-γ when combined with TNF on RASF, we were interested how RASF treated with these cytokines regulate B cell survival in co-cultures. We used murine B cells isolated from spleens as they do respond to human BAFF and CD40L, but only minimally to human TNF and not to human IFN-γ minimizing own effects of these cytokines^50–53^. Murine B cells cultured alone showed very little survival after 48 h, but survival was enhanced when conditioned medium of RASF was added, suggesting a pro-survival effect of a RASF-derived soluble factor. When murine B cells were cultured with RASF directly survival rates quadrupled compared to conditioned medium. This confirms results from Alasaleh *et al*. that showed that cell-cell contact is a prerequisite for class switch recombination in B cell RASF co-cultures and they identified BAFF as a major factor^37^. We also demonstrated that addition of BAFF alone or to co-cultures increased B cell survival and this was inhibited by the addition of the monoclonal anti-BAFF antibody belimumab. Although belimumab was considered to only block soluble BAFF, subsequent studies found that this antibody also inhibits membrane-bound BAFF^54^, and there are hints that inhibition of membrane BAFF might also be relevant in a clinical setting^55^. An effect on membrane bound BAFF might explain its inhibitory effect on B cells cultivated with unstimulated RASF, as they produce no soluble BAFF but do display membrane-bound BAFF under these conditions. We identified IFN-γ as the sole inducer of soluble BAFF in SF and therefore tested whether RASF stimulated with this cytokine increase B cell survival. Indeed, IFN-γ-stimulated RASF enhanced murine B cell survival and this effect was inhibited by belimumab, suggesting an influence of BAFF. Since membrane-bound BAFF was not modified by IFN-γ, soluble BAFF might be important as it is induced by IFN-γ. While TNF-stimulated RASF did not increase B cell survival, the combination of TNF and IFN-γ reduced survival compared to treatment with each cytokine alone. This was puzzling, since this combination was the strongest inducer of membrane-bound BAFF which is supposed to be indispensable for B cell survival^12^. However, RASF not only support B cell survival by BAFF but they also express other proteins involved in regulating B cell function. Another regulator of B cell survival, vascular cellular adhesion molecule 1 (VCAM-1), which is the cellular receptor for integrin α4β1 on B cells, is expressed in RASF^56^. Interestingly, we found TNF and to a lesser extent TNF/IFN-γ to up-regulate cell-bound VCAM-1, but only the combination strongly induced soluble VCAM-1. This suggests that IFN-γ when combined with TNF induces the shedding of VCAM-1 possibly by regulating a specific enzyme e.g. ADAM17^23^. Soluble VCAM-1 might then compete with membrane-bound VCAM-1 on RASF inhibiting cell-cell contact and thereby diminishing the pro-survival effect of RASF. Another cytokine that regulates B cell function is TGF-β and it is produced by RASF^57^. We detected the membrane-bound form on RASF, which was up-regulated by TNF and even more so by the combination of TNF and IFN-γ. TGF-β regulates many aspects of B cell function from Ig production to apoptosis and an up-regulation on RASF might reduce B cell survival^26^.

In addition to RASF, we also analyzed SF from patients with osteoarthritis (OA) as a non-chronically inflamed control. In all assays, we didn’t find any difference between OASF and RASF (data not shown). However, in vivo this might be completely different, since high levels of pro-inflammatory cytokines (e.g. TNF, IFN-γ) are only found in RA but not OA synovial tissue^58^. Hence, the in vitro observed up-regulation of receptors and cytokines of the TNF family in response to TNF/IFN-γ likely occurs in RASF exclusively.

## Conclusion

In this study we demonstrated synergistic effects of IFN-γ and TNF on the expression of TNF family member receptors, associated cytokine receptors and RASF proliferation. In contrast, cytokine production was negatively regulated by the combination of TNF and IFN-γ: While BAFF production was inhibited by TNF, IL-6 and IL-8 production was reduced by IFN-γ. Similarly, survival was increased by TNF or IFN-γ-stimulated RASF but reduced by the combination of both cytokines. These findings along with the shedding of VCAM-1 and the up-regulation of TGF-β by the combination of IFN-γ and TNF confirm a partial anti-inflammatory effect of IFN-γ in a TNF-driven pro-inflammatory environment in RA. In fact, it has already been shown that IFN-γR knockout mice show exacerbated arthritis^11^. Interestingly, in lupus erythematodes (SLE), IFN-γ is a prerequisite for disease development and this might be due to the cell type mainly responsible for disease initiation^59^. While lupus is mainly driven by the activity of autoreactive B cells, RA is dependent on T cell activation and innate mechanisms^60,61^. This is also evident in the therapeutic approach of both diseases, where RA profits from TNF inhibition, while SLE is combatted by B cell-depleting agents, like belimumab, whereas anti-TNF treatment exacerbates or even induces SLE. IFN-γ also induced soluble VCAM-1 when combined with TNF, which might, similar to soluble ICAM-1 alter leucocyte recruitment dependent on cell type and inhibit B cell attachment by disrupting VCAM1/integrin α4β1 interactions^62,63^. Additionally, increased membrane-bound TGF-β under the same culture conditions might not only alter B cell but also T cell function und support T-reg generation leading to an overall anti-inflammatory phenotype^64^.

## Materials and Methods

### Patients

28 patients with long-standing RA fulfilling the American College of Rheumatology revised criteria for RA^65^ were included in this study. The RA group comprised of 21 females and 7 males with a mean age of 63.6 years ± 7.6 years and 67.3 ± 9.8 years, respectively. C-reactive protein was 21.9 mg/dl ± 33.4 mg/dl for females and 67.7 mg/dl ± 61.4 mg/dl for males. 20 out of 28 patients received non-steroidal anti-inflammatory drugs, 20 out of 28 glucocorticoids, 5 out of 28 methotrexate, 1 out of 28 sulfasalazine, 3 out of 28 biologicals and 1out of 28 JAK inhibitor. All patients underwent elective knee joint replacement surgery, and they were informed about the purpose of the study and gave written consent. The study was approved by the Ethics Committees of the University of Düsseldorf (approval number 2018-87-KFogU) and Regensburg (approval number 15-1 01-021). We confirm that all experiments were performed in accordance with relevant guidelines and regulations.

### Antibodies and cytokines

All antibodies used in this study are listed in Table 1, cytokines are listed in Table 2. Antibodies used in this study (Table 1):

**Table 1:** Antibodies used for flow cytometry.

Vendor	Catalogue no.	Target	Fluorophore
Miltenyi	130-102-643	CD256 (APRIL)	APC
Miltenyi	130-105-822	CD266 (Fn14)	PE
Miltenyi	130-100-662	CD267 (TACI)	PE
Miltenyi	130-097-656	CD268 (BAFF-R)	FITC
Miltenyi	130-118-975	CD269 (BCMA)	APC
Miltenyi	130-096-654	CD106 (VCAM-1)	FITC
Miltenyi	130-114-133	CD154 (CD40L)	PE
Miltenyi	130-099-931	CD119 (INF-γR)	FITC
Miltenyi	130-105-153	CD295 (ObR, Leptin receptor)	PE-Vio770
Miltenyi	130-096-573	Membrane TGF-β	APC
ebioscience	12-9017-42	CD257 (membrane BAFF)	PE

**Table 2:** Cytokines used for stimulation of RASF.

Vendor	Catalogue no.	Cytokine
Peprotech	300-01 A	TNF
Peprotech	300-02	INF-γ
R&D	7537-BF	BAFF
R&D	5860-AP	APRIL
R&D	1090-TW	TWEAK

Antibodies were used in a 1:11 dilution as recommended by the supplier. Cytokines used in this study (Table 2):

### Synovial fibroblast and tissue preparation

Samples from RA synovial tissue were collected immediately after opening the knee joint capsule and tissue was prepared for cell isolation thereafter^66^. In brief, excised synovial tissue was cut into small fragments, minced and treated with liberase (Roche Diagnostics, Mannheim, Germany) at 37 °C overnight. The cell suspension was filtered (70 µm) and centrifuged at 300 g for 10 min. After that, the pellet was treated with erythrocyte lysis buffer (20.7 g NH_4_Cl, 1.97 g NH_4_HCO_3_, 0.09 g EDTA ad 1 l H_2_O) for 5 min, recentrifuged for 10 min and then resuspended in RPMI-1640 (Sigma Aldrich, St. Louis, USA) with 10% FCS. After overnight incubation, RPMI medium was replaced with fresh medium to wash off dead cells and debris.

### Stimulation of RA synovial fibroblasts

6000 cells were seeded onto 96 well microtiter plates, grown for three days and were then stimulated with either TNF (0.1–10 ng/ml), IFN-γ (0.1–10 ng/ml) or a combination (IFN-γ 10 ng/ml + TNF 10 ng/ml) for 72 h (in RPMI medium containing 2% FCS to minimize proliferation; for all assays). After that, cells or supernatants were used for flow cytometry (cells) and ELISA (supernatants).

### IL-6, IL-8, sVCAM-1, sCD40L, sTACI, sBCMA, BAFF and APRIL ELISA

Cell culture supernatants were used for ELISAs 72 h after addition of respective stimulants. Tests were conducted as described by the supplier (BD, OptEIA, Heidelberg, Germany (IL-6, IL-8) and R&D/Biotechne, Wiesbaden, Germany (sVCAM-1, sCD40L, sTACI, sBCMA, APRIL, BAFF)). Inter- and intraassay coefficient of variation was below 10%.

### Proliferation of RASF

Proliferation was assessed by the cell titer blue viability assay (promega). We analyzed proliferation in RPMI medium containing only 2% FCS. This was important since FCS contains up to 28 µM lysophosphatidic acid (LPA). LPA has been reported to induce proliferation, calcium mobilization and IL-8 production in a wide variety of cell types^67,68^ and therefore, we minimized this influence by only using 2% FCS.

### Flow cytometry

Data were acquired on a MACSQuant analyzer 10 (Miltenyi, Bergisch Gladbach, Germany). After detachment of RASF with accutase (Life-Technologies/Thermo, #A11105–01) at 37 °C for 20 min, RASF were incubated with primary labelled antibodies for 10 min at 4 °C in PBS/1% BSA and analyzed thereafter. Cells were single-stained with respective antibodies.

### Isolation of murine B cells

Murine B cells were isolated from spleens using the B Cell Isolation Kit mouse (miltenyi #130-104-443) according to the suppliers’ instructions.

### Animals

Male DBA/1 J mice (6–8 weeks old) were purchased from Janvier (France). They had unrestricted access to chow and water. Five animals lived in one cage, and they were acclimated to the environment for one week before commencement of experiments. The University of Düsseldorf approved all experiments according to institutional and governmental regulations for animal use (project: O57/15). All procedures were performed in accordance with the German Animal Welfare Act and the European Directive 2010/63/EU on the protection of animals used for scientific purposes.

### RASF co-culture with murine B cells

Co-culture experiments were performed in 48 well plates (Cellstar, Greiner bio-one, Kremsmünster, Austria). In brief, 20.000 RASF were seeded in 750 µl RPMI-1640 (Sigma-Aldrich) and grown for 72 h. Then, 250.000 isolated murine B cells were added for 48 h and cells were stimulated with cytokines as indicated. After that, B cell survival and apoptosis were assessed by Annexin V/propidium iodide staining.

### Apoptosis assay

B cell survival and apoptosis were assessed using the FITC Annexin V assay kit (BD biosciences, Heidelberg, Germany) according to manufacturers’ instructions.

### Statistical analysis

Statistical analysis was performed with SigmaPlot 13 (Systat Software Inc., San Jose, USA) and SPSS 25 (IBM, Armonk, USA). The statistic tests used are given in the figure legends. When data are presented as box plots, the boxes represent the 25th to 75th percentiles, the lines within the boxes represent the median, and the lines outside the boxes represent the 10th and 90th percentiles. When data are presented as line plots, the line represents the mean and error bars are depicted as standard error of mean. The level of significance was p < 0.05.
